# The Clinical Significance of Adsorption Phenomena

**Published:** 1920-12

**Authors:** Oliver C. M. Davis

**Affiliations:** Lecturer in Chemistry and Materia Medica in University of Bristol; Physician to Out-Patients, Bristol Royal Hospital for Sick Children and Women


### THE CLINICAL SIGNIFICANCE OF ADSORPTION PHENOMENA.

BY

OLIVER C. M. DAVIS, M.D. Bristol.

*Lecturer in Chemistry and Materia Medica in University of Bristol; Physician to Out-Patients, Bristol Royal Hospital for Sick Children and Women.*

---

THE object of the present paper is to show that many of the reactions which take place in the body during disease exhibit characteristics which are closely parallel to the phenomena which have been grouped together under the designation of adsorption or sorption.

The phenomena of adsorption have given rise to much discussion during the past century, and a vast amount of research has been carried out with the object of throwing light upon the mechanisms involved.

A few general remarks will first be made with reference to these phenomena before entering into a more specialised discussion, and as a typical example of adsorption may be cited the action of various types of carbon upon solutions of iodine in different solvents. Under favourable conditions the colour of the iodine solution diminishes in intensity immediately the solid carbon is brought into contact with this solution.

Various theories have been brought forward as to the nature of this action. Freundlich,[1] on the one hand, regarded the phenomenon as exclusively a condensation on the surface, whereas Travers[2] considered that adsorption consists exclusively of a more or less complete "absorption" into the interior of the solid.

A series of experiments on adsorption was carried out by the writer some years ago,[3] and will be briefly referred to here. The investigation consisted of a long series of experiments demonstrating the action of various types of carbon on standard solutions of iodine in different solvents.

It was clearly shown that " adsorption " may be accompanied by " absorption," and that in addition to a rapid condensation on the surface there is a slow diffusion inwards which goes on for long periods of time.*

At the time of these experiments certain of the tubes containing carbon, solvent, and iodine hermetically sealed up were left over, and various intervening matters prevented the work being continued till recently, when the tubes were opened and their contents examined under the supervision of Professor James W. McBain.[5]

The following table well illustrates the gradually increasing amounts of iodine taken up by the carbon during the prolonged period of time.

" a " is the original amount of iodine (1.2685 gm.) in 100 c.c. of the solvent ; " a—x " is the number of grammes of iodine left in solution after shaking with the carbon ; " m " is the weight of carbon (2 gm.) shaken with 100 c.c. of solution ; " x/m " therefore indicates the number of grammes of iodine sorbed by one gramme of carbon.

*Sorption by Animal Carbon of Iodine dissolved in Toluene.*

| Time | x/m. | a—x |
|---|---|---|
| 5 minutes | 0.229 | 0.809 |
| 15 ,, | 0.237 | 0.793 |
| 30 ,, | 0.245 | 0.777 |
| 1 hour | 0.265 | 0.736 |
| 2 hours | 0.293 | 0.682 |
| 5 ,, | 0.299 | 0.669 |
| 24 ,, | 0.315 | 0.637 |
| 5 days | 0.327 | 0.613 |
| 24 ,, | 0.379 | 0.509 |
| 11 years | 0.509 | 0.250 |

* McBain[4] has introduced the term " sorption " as a generic and non-hypothetical term for the two phenomena here mentioned, which frequently occur together.

After eleven years there was only the faintest trace of hydriodic acid present in the tube, showing that the diminution in concentration of the iodine solution cannot be ascribed to chemical action between solvent and solute.

The next table shows similar results in the case of iodine dissolved in benzene :—

| Time. | x/m. | a—x. |
|---|---|---|
| 3 days | 0.328 | 0.611 |
| 10 ,, | 0.339 | 0.589 |
| 24 ,, | 0.386 | 0.495 |
| 11 years | 0.561 | 0.146 |

Another important result of this research was to demonstrate conclusively that adsorption is a specific action depending here on the nature of the carbon. This is shown by the following table :—

*2 grammes Carbon to 100 c.c. solution of Iodine in Toluene.*

| *Time, 3 days.* | x/m. |
|---|---|
| Cocoanut Carbon | 0.062 |
| Sugar Carbon | 0.299 |
| Animal Carbon | 0.329 |

It is especially important to recognise two essential differences between chemical reactions and adsorption phenomena. In chemical reactions the position of an equilibrium set up is directly proportional to the concentrations of the reacting substances, and in dilute solution reaction becomes slower, whereas in the case of adsorption a nearly quantitative reaction occurs with very low concentrations, and such quantitative adsorption is instantaneous.

The result of these two factors is that a substance circulating in extreme dilution might be quantitatively adsorbed by specialised cells coming in contact with the circulating fluid, and be deposited on these cells in a state of comparatively high concentration. This is of special significance when it is remembered that a layer of adsorbed material only one molecule deep may alter all the properties of the solid so covered.[6]

Experience of that vast field of chemical reaction known as catalysis has demonstrated that these adsorption layers are the seat of enormously enhanced chemical reactivity. Thus in many important industrial reactions, such as the manufacture of sulphuric acid from sulphur dioxide and oxygen by the contact process, it has been shown that the reaction, otherwise perhaps undetectable, is instantaneous on the surface of the proper catalyst.

This is doubtless due to the very high local concentration of the reacting substances, which at the same time are fully exposed to the effects of the chemical and residual affinity of the surrounding material.

Such considerations as these, coupled with the results of the adsorption experiments with iodine, have led the writer to believe that there is a connection between adsorption phenomena and certain pathological processes. The work of Moore and Roaf[7] gives support to this belief, since these workers made laboratory experiments with anæsthetics upon brain tissue, etc., and concluded "that anæsthetics form unstable compounds or aggregates with the proteids of the tissue cells, and that anæsthesia is due to a paralysis of the chemical activities of the protoplasm as a result of the formation of such aggregations."

The subject is extremely complex, and as the toxic substances which will be referred to have not as yet been isolated in a pure condition, satisfactory experiments with them have been impossible up to the present. It must, however, be pointed out in such cases that unless investigators commence their research with a working hypothesis, progress must of necessity be extremely slow. It is obvious that experiments *in vitro* can never replace practical experiments with animals or clinical observations, but can only be used to support evidence obtained from other sources.

We are undoubtedly justified in carrying out laboratory experiments with substances which are readily adsorbed, and which can be estimated quantitatively with great accuracy, and in endeavouring to draw conclusions from such results which support or controvert various theories with regard to the mechanism of pharmacological action.

For this reason the experiments with carbon and iodine above described would appear to be of great value. In the writer's opinion there is clinical evidence to show that at any rate one of the earlier factors concerned in the action of certain toxins is adsorptive action. It would not be profitable at this stage of our knowledge to mention any considerable number of such probable cases, but a few will be noted. In the first place, those who have clinical experience with children are well aware of the alarming symptoms which may rapidly follow intestinal stasis, namely pain, drowsiness even approaching coma, and pyrexia or hyperpyrexia. In the majority of cases the administration of a mercurial purge modifies all the symptoms within a few hours. The symptoms point to a temporary alteration in the function of certain cerebral cells, and a feasible explanation is that this is due to the action of an alimentary toxin. That this toxin is chemically combined with the nervous tissue seems highly improbable, since the symptoms are so rapidly modified by drugs which presumably act by indirectly reducing the " active mass " and accumulation of the toxin by cutting off its source of supply, *i.e.* by bringing about an evacuation of decomposing material from the bowel.

That the toxin is adsorbed by the nervous tissue during the active period is a far more tenable theory than that of chemical action. In the days before the science of surgery had reached its present stage lardaceous disease or amyloid degeneration was far more common than at present. Here we presumably have small quantities of a toxin slowly but progressively poured out into the tissues, finally producing definite chemical changes in the cell units. By active treatment these degenerations may be prevented in the early stages, quite probably because purely chemical change is preceded by adsorption phenomena, at which stage appropriate treatment has a chance of removing the toxin and stopping its production. Still more striking is the case of the late cerebral lesions of syphilis. Here it may be assumed that the toxin thrown out by the Treponema pallidum in minute quantities is adsorbed by the cerebral tissues, and if not removed during early stages finally brings about histological changes in the cells.

There is one other type of disease in which the writer ventures to suggest that the phenomena of adsorption may be the main cause of the observed symptoms, namely the so-called "functional" nervous diseases.

At the present time the classification of diseases into "functional" and "organic" is becoming more and more unsatisfactory, as it is quite obvious that the physical properties of the constituent cells of the body may be profoundly modified without giving rise to any demonstrable histological change, the only evidence of such modification of properties being an observable disturbance in function. Such a modification of the physical properties of cells could certainly be brought about by an adsorption of toxins.

The fact that adsorption is a specific action appears to be of great importance if we assume that certain drugs or toxins undergo adsorption as previously stated by specialised tissues.

It is further suggested here that the two factors observed in the case of adsorption of iodine by carbon may also be present in the case of toxin action. A simple "adsorption"

or surface condensation would explain the rapid action of such toxins as those generated in the alimentary canal. If not removed these " adsorbed " substances would doubtless be slowly and progressively " absorbed," finally bringing about dire results, as in the case of lardaceous disease and syphilis previously referred to.

To summarise, it is here suggested that the action of toxins may take place in several stages, the first stage consisting of a condensation on the surface of specialised cells by adsorptive action. In this concentrated condition the toxins would exert a profound influence on the activities of the cells, and if not removed the second stage of diffusion inwards would be attained, accompanied or succeeded by definite chemical reactions bringing about histological changes.

REFERENCES.

1. Freundlich, *Habilitationschrift*, Leipzig, 1906.
2. Travers, " Adsorption and Occlusion," *Proc. Roy. Soc.*, 1906.
3. O. C. M. Davis, " The Adsorption of Iodine by Carbon," *Trans. Chem. Soc.*, 1907.
4. J. W. McBain. *Zeit. physik. Chem.*, 1909,
5. J. W. McBain, " Sorption of Iodine by Carbon," *Trans. Faraday Soc.*, 1919.
6. Langmuir, " Adsorption of Gases on Plane Surfaces," *J. Am. Chem. Soc.*, 1918.
7. Moore and Roaf, " The Physical and Chemical Properties of Solutions of Chloroform," *Proc. Roy. Soc.*, 1905.

